# Risk of Dementia among Veterans Experiencing Homelessness and Housing Instability

**DOI:** 10.1111/jgs.18680

**Published:** 2023-12-06

**Authors:** Jill S. Roncarati, Frank DeVone, Christopher Halladay, Jack Tsai, Eric Jutkowitz

**Affiliations:** 1 U.S. Department of Veterans Affairs, Center for Healthcare Organization & Implementation Research (CHOIR), VA Bedford Healthcare System, Bedford, Massachusetts, USA; 2 Department of Health Policy and Management, Harvard T.H. Chan School of Public Health, Boston, Massachusetts, USA; 3 Boston Health Care for the Homeless Program, Boston, Massachusetts, USA; 4 Center of Innovation in Long Term Services and Supports, Providence VA Medical Center, Providence, Rhode Island, USA; 5 U.S. Department of Veterans Affairs, National Center on Homelessness Among Veterans, Tampa, FL, USA; 6 University of Texas Health Science Center at Houston, School of Public Health, San Antonio Campus, TX 78229, USA; 7 Department of Health Services, Policy & Practice, Brown University School of Public Health, Providence, Rhode Island, USA

**Keywords:** Veterans experiencing homelessness, housing instability, housing insecurity, Alzheimer’s Disease, Dementia, Dementia related diseases, permanent supportive housing

## Abstract

**Background:**

In the United States, nearly 85,000 Veterans experienced homelessness during 2020, and thousands more are experiencing housing instability, representing a significant proportion of the population.^1^ Many Veterans experiencing homelessness are aging and have complex co-occurring medical, psychiatric, and substance use disorders. Homelessness and older age put Veterans at greater risk for age related disorders, including Alzheimer’s disease and related dementias (ADRD).

**Methods:**

We examined the rate of ADRD diagnosis for Veterans experiencing homelessness and housing instability compared to a matched cohort of stably housed Veterans over a nine-year period using Cox Proportional Hazard Models.

**Results:**

In the matched cohort, 95% (n=88,811) of Veterans were men, 67% (n=59,443) were White and were on average 63 years old (SD=10.8). Veterans with housing instability had a higher hazard of 1.53 (95% Confidence Interval (CI) 1.50, 1.59) for ADRD compared to Veterans without housing instability.

**Conclusions:**

Veterans experiencing housing instability have a substantially higher risk of receiving an ADRD diagnosis than a matched cohort of stably housed Veterans. Health systems and providers should consider cognitive screening among people experiencing housing insecurity. Existing permanent supportive housing programs should consider approaches to modify wrap-around services to support Veterans experiencing ADRD.

## INTRODUCTION

An estimated 1,253,000 people experienced homelessness during 2020 in the United States (US).^1^ Approximately 33,129 are Veterans, representing 7% of all homeless adults.^2^ Thousands more Veterans experience housing instability and are at-risk for homelessness every year. Veterans experiencing homelessness and housing instability have complex co-occurring medical, psychiatric, and substance use disorders.^3,4^ Similar to the general US population, the homeless population is rapidly aging.^5^ Almost 60% of Veterans experiencing homelessness are 50 years old or older.^6,7^ Furthermore, it is estimated that the number of homeless individuals 55 years and older will increase by almost a third in just the next four years, from 170,000 today to 225,000.^8^

As the homeless population ages, they are at-risk for geriatric conditions. Alzheimer’s disease and related dementias (ADRD) is one of the most challenging aging related diseases.^9^ ADRD is costly and there is no cure for the disease.^10^ Over the long course of the disease people lose the ability to live independently and require assistance to perform instrumental and basic activities of daily living.^11^ Most people living with ADRD receive a substantial amount of care from family caregivers.^12^ However, older homeless adults usually lack traditional social supports such as a spouse or child caregiver that many older Americans rely on to age in the community.^13^

Most older adults with dementia are either undiagnosed or unaware of the diagnosis.^14^ Despite an underdiagnosis, the prevalence of ADRD is substantially higher among Veterans experiencing homelessness (3.60%) and housing instability (13.48%), defined as at-risk of experiencing homelessness due to extreme poverty, inadequate housing, or failure to make mortgage/rent payments or are uncertain about the ability to make payments, compared to stably housed Veterans (3.04%).^15^ Several factors may contribute to the higher prevalence of ADRD among homeless and housing insecure Veterans compared to stably housed Veterans. First, African American and Hispanic Veterans are disproportionately represented among the homeless population.^16^ Simultaneously, African Americans are twice as likely and Hispanics are one-and-a-half times more likely than Whites to be diagnosed with ADRD.^9^ Second, comorbidities common in the homeless and housing insecure population, such as alcohol and/or substance use disorders, depression, and post-traumatic stress disorder, are also risk factors for ADRD.^17^ Third, symptoms of ADRD may cause economic instability such as an inability to manage finances and work and loss of housing. For example, in a recent analysis we found Veterans who received a diagnosis of ADRD, especially at ages younger than 69 years old, had an increased risk of becoming homeless.^18^ ADRD among the housing insecure population presents a new set of challenges for the long-term care system including how to help these individuals age safely in a community and determining the optimal time to move to a nursing home.^19^

An important next step to inform policy and programs serving housing insecure populations is to understand whether Veterans experiencing homelessness or housing instability age into ADRD at an increased rate compared to stably housed Veterans. To address this gap, we conducted a 9-year retrospective matched cohort study of Veterans who were Veterans Health Administration users with the primary aim of determining the rate of ADRD diagnoses for Veterans experiencing instability compared to Veterans with housing stability.

## METHODS

### Study Data and Sample

We examined the rate of ADRD diagnosis by housing status (housing instability compared stably housed) from January 1^st^, 2011 to December 31^st^, 2019. To accomplish this objective, we identified Veterans without ADRD prior to 2011 and who experienced housing instability for the first time in 2010.

**Figure 1.**
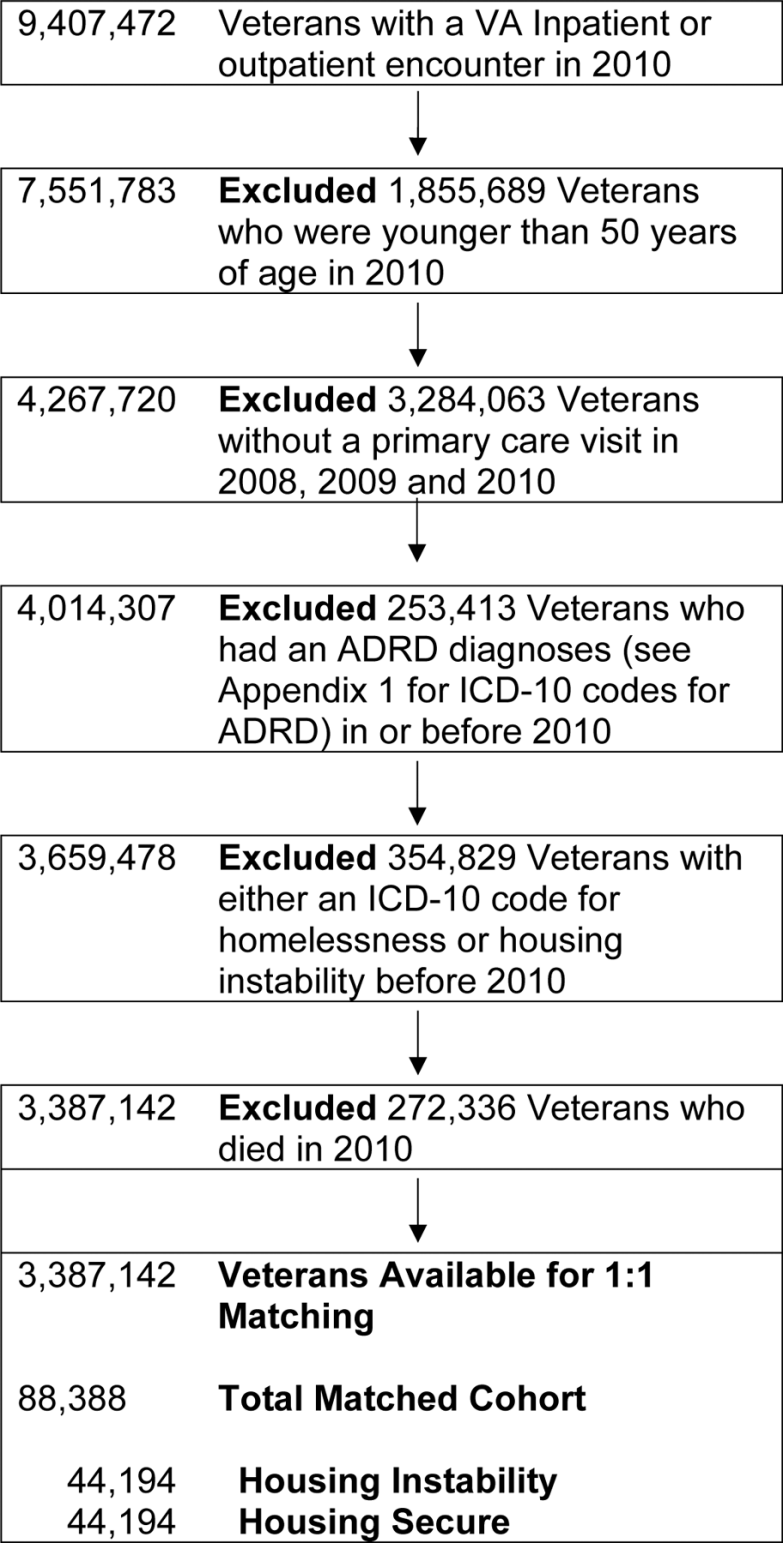
Flow Diagram for Cohort Selection

To create the analytic cohort, we queried the Veteran Affairs (VA) Corporate Data Warehouse to identify all Veterans who had a VA paid health care encounter in 2010 (n=9,407,472). First, we excluded Veterans who were younger than 50 years of age, as their risk of ADRD is low (n=1,855,689). Second, we excluded Veterans without a primary care visit in 2008, 2009 and 2010 (n=3,284,063) because without a VA encounter we could not know if they were diagnosed with ADRD or not. Third, we excluded Veterans with an International Classification of Diseases 10^th^ Revision (ICD-10) code for ADRD using the Chronic Conditions Data Warehouse definition for ADRD^20^ (see Supplementary Table S1a for ICD-9 and 10 codes) before 2011 (n=253,413), Veterans with ICD-10 codes for homelessness or housing instability before 2010 (n=354,829), and Veterans who died in 2010 (n=272,336). Among this cohort, we determined whether a Veteran had a new diagnosis of homelessness or housing instability in 2010 using ICD-10 codes^15^ (see Supplementary Table S1b for 10 codes; n=44,194). All other Veterans were categorized as having housing stability (n=3,342,948; Figure 1).

### Dependent Variable

We followed all Veterans from 2011 to the end of 2019 to determine whether they ever received a diagnosis of ADRD. Death or not receiving an ADRD diagnosis were censoring events. The median follow-up time was 3,286 days in both groups; however, among Veterans experiencing housing instability, the lower bound interquartile range (Q1) was shorter at 1,224 days as compared to 1,740.25 days.

### Independent Variables

We controlled for measures associated with housing and ADRD. The independent variables obtained from the Corporate Data Warehouse were age, race, marriage status, Veteran enrollment priority status, combat Veteran, and whether they lived in a rural area.^21^ We used the rural-urban commuting area (RUCA) codes, which are used by the Office of Management and Budget (OMB), to define county-level metropolitan and micropolitan areas.^21^ Veterans are given a priority status to determine cost-sharing, level of need and access to services. We created a binary variable in our study for Veterans in priority group one, who are Veterans with a service-connected disability that is ≥50% or more disabling, or who unable to work due to a service-connected disability. We used priority status as an indicator of disability and need and who are at great risk of negative health outcomes.^22^ We used the Agency for Healthcare Research and Quality’s Clinical Classification Software codes to determine whether a Veteran had diagnoses in 2010 for rheumatic disease, renal disease, liver disease, diabetes mellitus, hypertension, heart failure, pulmonary disease, valvular disease, cerebrovascular accident, traumatic brain injury, alcohol use disorder, drug use disorder, depression, posttraumatic stress disorder, or psychoses.^23^ Finally, we determined whether a Veteran had at least one hospice encounter or nursing home stay in 2010.

### Statistical Analyses

We compared the demographic characteristics of Veterans by their housing status in 2010. The standard or raw mean difference (SMD/RMD) were ≥0.10 for most characteristics. Therefore, we used propensity scores one-to-one nearest neighbor matching to create a matched cohort of Veterans with and without housing instability, using all covariates (Table 1) with a SMD/RMD ≥0.10.^24^ Specifically, we matched on age, race, marriage status, Veteran enrollment priority status, combat Veteran status, rurality, rheumatic disease, renal disease, liver disease, diabetes mellitus, hypertension, heart failure, pulmonary disease, valvular disease, cerebrovascular accident, traumatic brain injury, alcohol use disorder, drug use disorder, depression, posttraumatic stress disorder, psychoses, hospice stay, and nursing home stay.

We estimated the unadjusted and adjusted rate of being diagnosed with ADRD using Cox Proportional Hazard Models on the matched cohort. The adjusted model controlled for all the independent variables obtained from the Corporate Data Warehouse (Table 1).^24^ In a sensitivity analysis, we censored observations from Veterans who were stably housed at the start of the study once they received a diagnosis of homelessness during follow up (n=2,156,742). Veterans were censored upon death in all versions of the analysis. Analyses were conducted using R version 4.1.2 with survival, MatchIt, and Survminer Packages.

## RESULTS

We matched 44,194 Veterans with housing instability to 44,194 Veterans with secure housing (Table 1). In the matched cohort 95% (n=88,811) of Veterans were men, 67% (n=59,443) were White, and the average age was 63 years old (SD=10.8). Nearly half (48% of Veterans with housing instability and 42% of securely housed Veterans) of the sample died during the study timeframe, Veterans were censored if this occurred. Co-morbidities were common (Table 1). Characteristics of the pre-matched cohort are given in Supplementary Table S2.

**Figure 2.**
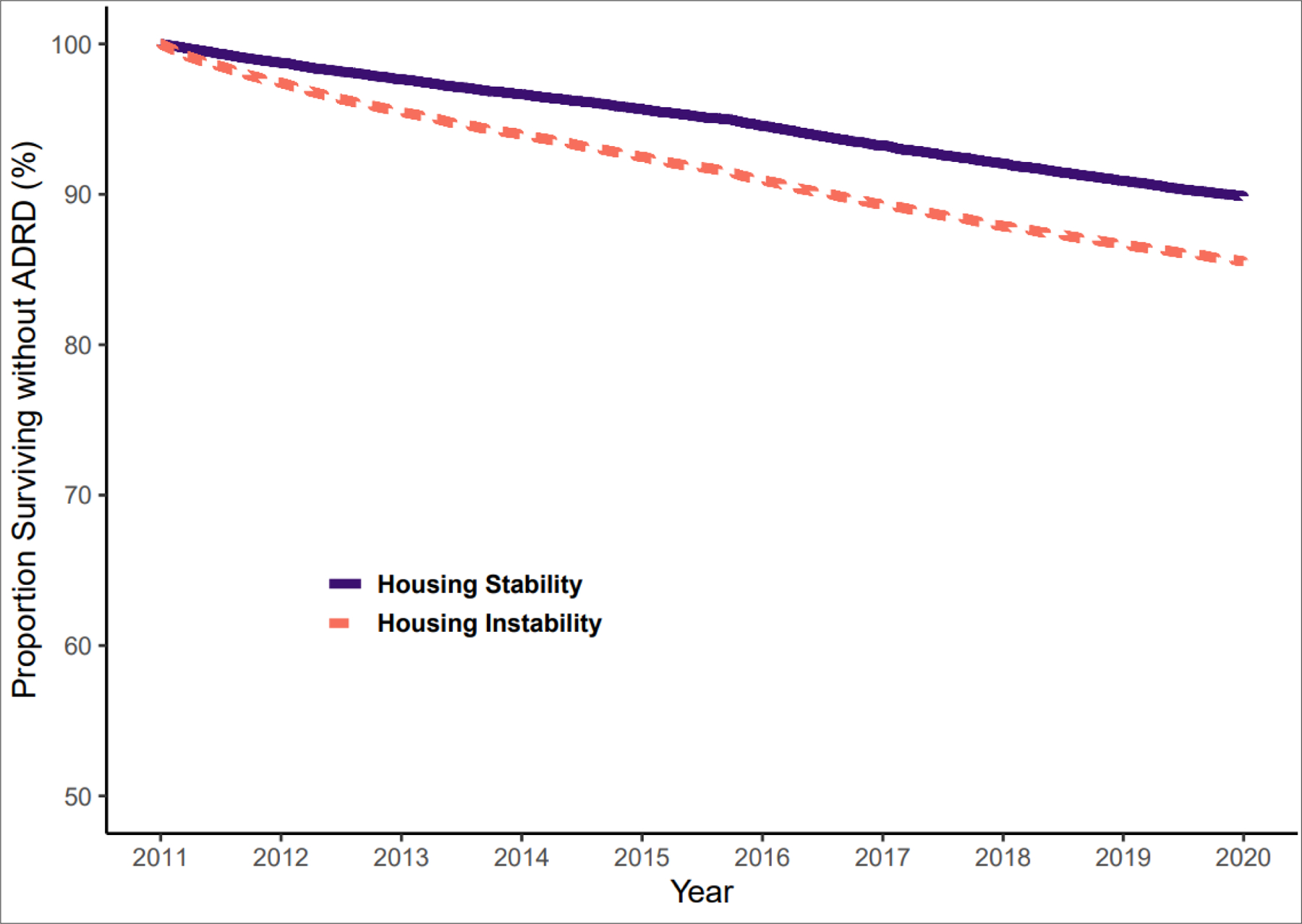
Kaplan-Meier Estimated Curves for Veterans Experiencing Housing Instability and Matched Controlled Veterans Housing stability: Veterans without ICD-10 codes for homelessness or housing instability. Housing instability: Veterans with an ICD-10 codes for homelessness or housing instability.

In the unadjusted model, Veterans with housing instability had a 1.33 (95% CI: 1.27 to 1.38) increase in the rate of ADRD compared to Veterans with housing stability (Table 2). In the adjusted model, Veterans with housing instability had a 1.41 (95% CI: 1.36 to 1.47) increase in the rate of ADRD compared to Veterans with housing stability. In sensitivity analyses that censored observations from Veterans who were stably housed at the start of the study once they received a diagnosis of homelessness during follow up, Veterans with housing instability continued to have a higher rate of ADRD (hazard of 1.53, 95% CI (1.50, 1.59)) than Veterans with housing stability. Figure 2 plots the risk of ADRD diagnosis from 2011 to 2019. By 2015, the midpoint of the study, 7.23% and 3.66% of housing insecure and housing stable Veterans had an ADRD diagnosis, respectively.

## DISCUSSION

We examined the risk of receiving an ADRD diagnosis in a population of housing instability and stably housed Veterans. Studies have found a higher prevalence of ADRD diagnoses for Veterans experiencing housing instability compared to Veterans who are stably housed,^15^ and an increased prevalence of ADRD in older homeless populations post-mortem.^25^ Although housing instability is associated with ADRD, there are limited data on the directionality of this association. That is, are Veterans with ADRD more likely to eventually become homeless or are Veterans who have experienced housing instability more likely to eventually develop ADRD? Our findings further add to the overall understanding of ADRD among Veterans who are homeless or experiencing housing instability. Key results include that Veterans experiencing homelessness or housing instability have a higher risk of eventually receiving an ADRD diagnosis. Combined with data that Veterans with ADRD are also more likely to eventually become homeless, our findings support a conclusion that the high prevalence of ADRD among Veterans experiencing housing instability is a combination of pathways that lead to a higher prevalence of ADRD among housing insecure Veterans.

These increases in the prevalence and incidence of ADRD among homeless populations,^15,18^ underscore a need to re-think models of supportive housing. The challenges of providing ADRD care for homeless populations are magnified by the lack of permanent housing and models of care that allow older clients to “age in place.” Much ADRD care is either provided in the community or home, usually by family members.^26^ Without a safe home environment, housing insecure populations with ADRD may need to rely on nursing home care sooner than stably housed populations. Nursing home care is costly and clinically may not be needed until later in the disease stage.^27^

These findings call for permanent supportive housing with wrap-around ADRD services. The U.S. Department of Housing and Urban Development-Veterans Affairs Supportive Housing (HUD-VASH) is a program providing permanent supportive housing for Veterans. HUD-VASH offers case management and some clinical support services. To support Veterans with ADRD in HUD-VASH, clinical services such as daily nursing visits for medication administration and daily home health aide visits for help with activities of daily living might need to be incorporated into the service delivery housing model. Linkages to supportive services that can help substitute for family caregiving include adult day health care and other opportunities for volunteering and giving purpose to life. Importantly, many of these options will require providing the Veteran with transportation.

Successful independent living for housing insecure Veterans with ADRD may require additional options, including federally funded housing vouchers for non-traditional locations like assisted living facilities. These options could be valuable tools to include in a housing continuum that aims to preserve independence. In addition, assisted living facilities are often considerably less expensive than nursing homes. Any program that places housing insecure Veterans in assisted living facilities would have to ensure vouchers covered the entire cost of a stay.

The VA has several innovative health delivery programs for homeless Veterans including the Health Care for Homeless Programs and VA Homeless Patient Aligned Care Teams. These tailored service delivery models utilize co-located and integrated multidisciplinary care teams.^28^ Medical and behavioral health providers share clinic space or see patients together and the teams are usually fully integrated into the existing health care system. The models of care focus on continuity and quality of care, essential elements in caring for this iterant and vulnerable population struggling to survive without the safety and stability of a home. These programs should consider incorporation of ADRD screening with proper follow-up and referrals into the routine care for aging individuals in their census. ADRD makes managing comorbidities challenging and may put greater pressure on providers. As a result, there is a need to incorporate specialized clinical training in the primary care setting in how to manage ARDR for a homeless-experienced population.

### Limitations

Our study had some limitations. We used ICD-10 codes to identify Veterans experiencing homelessness, housing instability, and ADRD, which are diagnoses codes reported by a provider in the VA administrative records. We limited our analysis to VA users to account for diagnosis bias. Nevertheless, we may have misclassified Veterans with infrequent VA encounters. We did not censor Veterans who may have stopped going to VA providers. Excluding Veterans without a primary care visit may inadvertently include people with ADRD but because they have not gone to primary care, they will not receive an ADRD diagnosis. The severity of the ADRD diagnoses were not known nor was the level of Veterans functional independence. Only VA administrative data were used to determine whether Veterans ever received an ADRD diagnosis. Future studies should consider the role of social determinants of health variables such as education and direct access to primary care, which are risk factors for ADRD and factors that affect who receives a diagnosis. We combined homeless and housing instability; but interventions created to support these two groups will likely be different. Programs for Veterans experiencing housing instability may focus on helping them age in their current home safely. For a homeless individual with ADRD, finding appropriate housing with supports would be imperative but also challenging given the scarcity of resources.

### Strengths

We stopped our study timeframe just before the COVID-19 pandemic to prevent the data from being skewed due to fewer visits and diagnostic testing during the height of the pandemic. We have detailed ICD and demographic information on all participants. The retrospective study design meant few, if any, Veterans were lost to follow up.

Our findings are generalizable from the Veteran experiencing housing insecurity population to the general homeless and housing insecure population at the needs for both are similar.^29–31^ Our findings are a call to action for permanent supportive housing programs and clinicians to incorporate ADRD support services and staff training. This would be to stave off nursing home admissions and bolster housing stability once a person experiencing homelessness received housing. Also thinking about housing as a continuum a person may need to move through as they become less independent but still do not need a nursing home is now imperative.

In conclusion, Veterans experiencing housing instability have a substantially higher risk of receiving an ADRD diagnosis than stably housed Veterans. Providers should consider targeted cognitive screening among older Veterans experiencing housing instability who have high-risk profiles. Permanent supportive housing with wrap-around services including medical, nursing, home health, and social services may delay the need for nursing home care. Additionally, using housing vouchers at assisted living facilities when independent living is no longer possible, is a practical approach to help older housing insecure Veterans with ADRD age safely into a community environment.

## Supplementary Material

Tab S1 Supplementary Table S1a. Alzheimer’s Disease and Related Dementias (ADRD) ICD-9 and ICD-10 Codes
Supplementary Table S2. Characteristics of pre-matched cohort for Veterans with and without housing instability in 2010

## Figures and Tables

**Table 1.** Characteristics of post-matched cohort for Veterans with and without housing instability in 2010

		Total Matched Cohort N=88,388 N (%)	Housing instability N=44,194 N (%)	Housing Secure N=44,194 N (%)	SMD/RMD
Age, mean (SD)		63.5 (10.8)	63.6 (10.9)	63.4 (10.7)	0.012
Gender	Men	83,811 (95)	42,270 (96)	41,541 (94)	0.075
	Women	4,577 (5)	1,924 (4)	2,653 (6)	0.075
Race	White	59,443 (67)	29,763 (67)	29,680 (67)	0.004
	Black	19,188 (22)	10,114 (23)	9,074 (21)	0.057
	Other	9,757 (11)	4,317 (10)	5,440 (12)	0.081
Not Married		55,310 (68)	28,173 (69)	27,137 (68)	0.008
Combat Veteran		9,532 (11)	4,829 (11)	4,703 (11)	0.009
Rurality		22,963 (26)	11,536 (26)	11,427 (26)	0.006
**Comorbidities, N (%)**					
Rheumatic Disease		1,670 (2)	871 (2)	799 (2)	0.012
Renal Disease		8,703 (10)	4,357 (10)	4,346 (10)	0.001
Liver Disease		6,390 (7)	3,213 (7)	3,177 (7)	0.003
Diabetes Mellitus		26,781 (30)	13,787 (31)	12,994 (29)	0.039
Hypertension		58,824 (67)	29,472 (67)	29,352 (66)	0.006
Heart Failure		8,819 (10)	4,384 (10)	4,435 (10)	0.004
Pulmonary Disease		21,607 (25)	10,743 (24)	10,864 (25)	0.006
Valvular Disease		3,863 (4)	2,035 (5)	1,828 (4)	0.023
Stroke		10,121 (11)	5,012 (11)	5,109 (12)	0.007
Traumatic Brain Injury		2,261 (3)	1,174 (3)	1,087 (2)	0.013
Alcohol Use Disorder		11,272 (13)	5,680 (13)	5,592 (13)	0.006
Drug Use Disorder		2,195 (3)	1,246 (3)	949 (2)	0.043
Depression		31,300 (35)	15,522 (35)	15,778 (36)	0.012
PTSD		13,523 (15)	6,772 (15)	6,751 (15)	0.001
Psychoses		22,147 (25)	10,971 (25)	11,176 (25)	0.011

* standardized or raw mean difference (SMD/RMD)

**Table 2.** Hazard Ratios for the Association Between Housing Instability and ADRD From Unadjusted and Adjusted Cox Regression Models

	Housing Instability vs. Housing Stability	
	Matched Cohort without Regression Adjustment	Matched Cohort with Regression Adjustment^b^
**Primary Analysis**	1.33 (1.27,1.38)	1.41 (1.36, 1.47)
**Sensitivity Analysis** ^a^	1.53 (1.50,1.59)	1.63 (1.56, 1.70)

a Censored Veterans who had a new diagnosis for housing instability during follow up.

b Adjusted for demographics, comorbidities, and hospice use and nursing home use in the year before the study.

## Key Points:

1. Veterans experiencing housing instability have a substantially higher risk of receiving Alzheimer’s disease or related dementia diagnosis than stably housed Veterans.
2. Veterans with Alzheimer’s disease or related dementia diagnosis are more likely to eventually become homeless.
3. The high prevalence and high incidence of Alzheimer’s disease or related dementia diagnosis among homeless Veterans underscore need to rethink supportive housing models.

## Box

**This paper matters** because it shows that Veterans who have experienced housing instability are more likely to be diagnosed with Alzheimer’s disease or a related dementia. Veterans who were stably housed at the start of the study and during the study received a diagnosis of homelessness had a higher rate of Alzheimer’s disease or related dementia diagnosis (hazard of 1.53, 95% confidence interval (1.50, 1.59)) than Veterans with housing stability.
