# Supplementary material for: Risk of Dementia among Veterans Experiencing Homelessness and Housing Instability
Source: J Am Geriatr Soc. Author manuscript; Available in PMC 2025 Feb 1. (PMC10922215; doi:10.1111/jgs.18680)
Supplement: Tab S1 — Supplementary Table S1a. Alzheimer’s Disease and Related Dementias (ADRD) ICD-9 and ICD-10 Codes Supplementary Table S2. Characteristics of pre-matched cohort for Veterans with and without housing instability in 2010 [file NIHMS1945800-supplement-Tab_S1.pdf]

**Supplementary Table S1a.** Alzheimer's Disease and Related Dementias (ADRD) ICD-9 and ICD-10 Codes	
**ICD-10 Code**	**Description**
F01.50	Vascular dementia without behavioral disturbance
F01.51	Vascular dementia with behavioral disturbance
F02.80	Dementia in other diseases classified elsewhere without behavioral disturbance
F02.81	Dementia in other diseases classified elsewhere with behavioral disturbance
F03.90	Unspecified dementia without behavioral disturbance
F03.91	Unspecified dementia with behavioral disturbance
F04	Amnestic disorder due to known physiological condition
G13.8	Systemic atrophy primarily affecting central nervous system in other diseases classified elsewhere
F05	Delirium due to known physiological condition
F06.1	Catatonic disorder due to known physiological condition
F06.8	Other specified mental disorders due to known physiological condition
G30.0	Alzheimer's disease with early onset
G30.1	Alzheimer's disease with late onset
G30.8	Other Alzheimer's disease
G30.9	Alzheimer's disease, unspecified
G31.1	Senile degeneration of brain, not elsewhere classified
G31.2	Degeneration of nervous system due to alcohol
G31.01	Pick's disease
G31.09	Other frontotemporal dementia
G94	Other disorders of brain in diseases classified elsewhere
R41.81	Age-related cognitive decline
R54	Age-related physical debility
**ICD-9 Code**	**Description**
331.0	Alzheimer's disease
331.11	Pick's disease
331.19	Other frontotemporal dementia
331.2	Senile degeneration of brain
331.7	Cerebral degeneration in diseases classified elsewhere
290.0	Senile dementia, uncomplicated
290.10	Presenile dementia
290.11	Presenile dementia with delirium
290.12	Presenile dementia with delusional features
290.13	Presenile dementia with depressive features
290.20	Senile dementia with delusional features
290.21	Senile dementia with depressive features
290.3	Senile dementia with delirium
290.40	Vascular dementia, uncomplicated
290.41	Vascular dementia, with delirium
290.42	Vascular dementia, with delusions
290.43	Vascular dementia, with depressed mood
290.0	Senile dementia, uncomplicated
294.10	Dementia in conditions classified elsewhere without behavioral disturbance
294.11	Dementia in conditions classified elsewhere with behavioral disturbance
294.20	Dementia, unspecified, without behavioral disturbance
294.21	Dementia, unspecified, with behavioral disturbance
294.8	Other persistent mental disorders due to conditions classified elsewhere
797	Senility without mention of psychosis

**Supplementary Table S1b.** Homelessness and Housing Instability ICD-10 Codes

ICD-10 Code	Description
Z59.0	Homelessness
Z59.1	Inadequate housing
Z59.3	Problems related to living in residential institution
Z59.5	Extreme poverty
Z59.8	Other problems related to housing and economic circumstances
Z59.9	Problem related to housing and economic circumstances, unspecified
Z62.21	Child in welfare custody
Z74.2	Need for assistance at home and no other household member able to render care
